# New categories of psychiatric disorders related to mild neuroinflammation-autoimmune psychosis, mild encephaliutis

**DOI:** 10.1192/j.eurpsy.2022.932

**Published:** 2022-09-01

**Authors:** K. Bechter

**Affiliations:** University Clinic Ulm, Psychiatry And Psychotherapy 2, Bezirkskrankenhaus Günzburg, Günzburg, Germany

## Abstract

**Introduction:**

The mild encephalitis hypothesis(ME) ( Bechter 2001, NPBR; updated Bechter 2013,Progr NP&BP) proposed that mild neuroinflammation triggered by infections, autoimmunity, trauma or toxicity (including from stress) might causally underly a spectrum of severe mental disorders (SMDs), especially disorders of the schizophrenic and affective spectrum.

**Objectives:**

The development from ME hypothesis to the new diagnoses of Autoimmune Psychosis (AP) and a subgroup of Autoimmune Encephalitis (AE) and beyond into future research is reviewed and discussed.

**Methods:**

Expert review

**Results:**

The subgroup of AE with exclusive or predominant psychiatric symptoms ( compare Graus et al 2016) and all cases of AP ( Pollak et al,Lancet Psychiatry 2020) match the previous poposed ME criteria. AE and AP can now successfully be treated in majority of cases by immune modulatory treatments. These new insights challenge both, the implementation of diagnosis and treatment into clinical reality and forthcoming research on the causality underlying severe mental disorders (SMDs). CSF studies showed in 50-70% of therapy resistant cases of affective and schizophrenic spectrum disorders some abnormalities compatible with mild neuroinflammation ( Bechter et al 2010, J Psych Res), recently confirmed in large patient samples from various university hospitals in Germany ( Endres et al 2018 aso., Rattay et al 2021, aso.).Also post mortem findings are compatible with ME hypothesis in a larger subgroup of SMDs. Open questions of new clinical categorization by refined grading of mild neuroinfalmmation by improved diagnostic methods appear increasingly required, which will be discussed ( Bechter Frontiers Psychiatry 2020).

**Conclusions:**

Mild neuroinflammation appears causally involved in SMD

**Disclosure:**

No significant relationships.

